# The Influence of Chlorhexidine Gluconate Dentine Pre-Treatment on Adhesive Interface and Marginal Sealing

**DOI:** 10.3390/medicina59020278

**Published:** 2023-01-31

**Authors:** Mihai-Octavian Boaru, Ionuț Tărăboanță, Simona Stoleriu, Sorin Andrian, Galina Pancu, Irina Nica, Irina-Georgeta Sufaru, Gianina Iovan

**Affiliations:** Faculty of Dental Medicine, Grigore T. Popa University of Medicine and Pharmacy, 16 Universitatii Str., 700115 Iasi, Romania

**Keywords:** microleakage, universal adhesive, chlorhexidine, matrix metalloproteinases, scanning electron microscopy SEM

## Abstract

*Background and Objectives*: The aim of this in vitro study was to evaluate the combined effect of a 2% chlorhexidine aqueous solution and a universal adhesive system applied in self-etch and etch-and-rinse strategies on the composite resin–dentin interface. *Materials and Methods*: Class V cavities were prepared on the facial and lingual surfaces of forty caries-free molars extracted for orthodontic reasons. The samples were randomly divided into two groups corresponding to the used etching protocol: I—etch-and-rinse; II—self-etch. In each tooth, one cavity was assigned for the control subgroups -IA (n = 20) and IIA (n = 20)—adhesive only, and the opposite cavity was pretreated with a 2% chlorhexidine solution—Gluco CHeX Cerkamed—subgroups IB (n = 20) and IIB (n = 20). Both sets of groups were restored using a universal adhesive system (Single Bond Universal Adhesive, 3M-ESPE) and a bulk-fill composite resin (Filtek One Bulk Fill Restorative, 3M-ESPE). The roots and the pulp tissue were then removed, and a needle connected to a perfusor with 100 mL saline solution was used for pulp pressure simulation with a hydrostatic pressure of 20 cm H_2_O. Cariogenic attack was simulated using a demineralizing solution for 3 days at a constant temperature of 25 °C. The teeth were then sectioned in a facial-lingual direction and the microleakages at the occlusal and cervical margins were registered and scored using an optical Carl-Zeiss AXIO Imager A1m microscope (Carl-Zeiss). The composite resin–dentin interface was analyzed using a SEM Vega Tescan LMH II. Statistical analysis was performed using the Kruskal–Wallis test with a significance level of *p* < 0.05. *Results*: Microleakage evaluation showed no significant differences among the study groups (*p* > 0.05). In subgroup IA, significant differences were recorded between occlusal and cervical margins (*p* < 0.05). *Conclusions*: Application of chlorhexidine on tooth substrate before using a universal bonding system in total etch or self-etch mode has no influence on the adhesive interface in the condition of cariogenic attack. The thickness of the adhesive resin layer seems to be less uniform when using chlorhexidine, but the morphological differences at the adhesive interface have no influence on the sealing capacity of the universal bonding system, regardless of the etching strategy.

## 1. Introduction

Dentin is a hard dental tissue composed of a mineralized collagen matrix, which does not have the ability to repair itself like bone tissue. Therefore, direct restorative treatment with resin adhesive is the most common possibility for its repair [1].

Modern adhesive resins for restorative therapy can form strong bonds of 30–50 MPa with dentin, but for a limited time [2]. The limited durability of this bond affects the restoration’s longevity [3]. Previous studies have shown that 75% of resin restorations fail as a result of the degradation of the interface between the adhesive resin and dentin, resulting in restoration loss, secondary caries or pulpal sensitivity [4,5]. The presence of a durable joint between the composite resin and dentin improves the strength of the restored tooth, provides an optimal sealing and increases the durability of the restoration [2,6].

The adhesion of composite resin to dentin can be achieved by using an etch-and-rinse (ER) or self-etch (SE) adhesive system which creates a hybrid layer at the interface between the dentin and the composite resin [7]. Both types of systems create a hybrid layer (HL) consisting of partially demineralized dentine impregnated with resin. The HL varies with the adhesive strategy in terms of layer thickness; in the etch-and-rinse technique, HL is thicker than the one observed in the self-etch strategy [8]. Resin also penetrates and polymerizes in the open dentinal tubules forming the resin tags [9].

HL can be degraded by the aqueous environment as a result of resin hydrolysis and deterioration of the demineralized collagen [10]. Residual water originates from the external environment, from the adhesive system when a water-based solvent is used, or from the intrinsic humidity of dentin [2]. Water might interact with the hydrophilic monomers, such as HEMA (2-Hydroxyethylmethacrylate), through hydrogen bonds or van der Waals forces [11,12], or inhibit the polymerization of the resin monomers and, later on, it might plasticize the polymers [12,13,14].

On the other hand, when the resin monomers do not fully infiltrate the demineralized dentin, the collagen fibers in the lower area of the HL remain exposed to degrading phenomena [1,15]. A type of endogenous proteases with a collagenolytic effect called matrix metalloproteinases (MMPs) can be found in the dentin matrices, and they are activated by the ER and SE adhesive systems [2]. MMPs can affect the HL structure by degrading the collagen matrix [16]. The desire to counteract these harmful effects on adhesion to dentin has required the adoption of several strategies based on the use of non-specific MMP inhibitors, such as chlorhexidine (CHX) [17]. According to recent studies, 0.1–2% chlorhexidine solutions exert an inhibitory effect on MMP-2, -8 and -9, thus reducing the degradation of the adhesive interface between composite resins and dentin [18,19,20].

A previous study concluded that CHX can reduce the immediate adhesion to dentin of both self-etch and etch-and-rinse techniques [21]. On the other hand, other studies showed that the use of CHX improves the bond strength to dentin for adhesives applied by the etch-and-rinse technique [22,23]. The conclusions from the literature are controversial because in the case of the etch-and-rinse technique, a discrepancy between the depth of demineralization and the depth of resin infiltration is incriminated [23].

Universal bonding systems were introduced with the purpose to obtain the adhesion of composite resins to many types of substrates in both strategies of application (ER and SE). A limited number of studies and lack of common opinion regarding the efficacy of these bonding systems in association with CHX is in the literature, especially regarding when they are applied in SE mode [24,25]. Moreover, in an oral cavity, the tooth-adhesive interface can be subjected to different challenging conditions (chemical, physical or biological) which might prone the adhesion to fail.

The aim of this in vitro study was to evaluate the effects of a 2% chlorhexidine aqueous solution on the composite resin–dentin interface when a universal adhesive system was applied in self-etch and etch-and-rinse strategies in the condition of cariogenic attack. The null hypothesis sustained the absence of significant changes of the composite resin–dentin interface morphology, and microleakage scores of the samples pre-treated with 2% chlorhexidine gluconate compared to samples that were not pre-treated.

## 2. Materials and Methods

The study was conducted in accordance with the Declaration of Helsinki and complied with all the rules imposed by the Ethics Commission of “Grigore T. Popa” University of Medicine and Pharmacy Iași (no. 54/01.03.2021).

### 2.1. Sample Preparation

The sample size was calculated using G * Power software (Heinrich-Heine Universität Düsseldorf, Düsseldorf, Germany) version 3.1.9.7. The chosen effect size of 0.3 is a medium effect according to Cohen classification. An alpha value of 0.05 and an 80% power were used. A total number of 79 samples was required for the study.

Forty molars extracted for orthodontic reasons were used in the study. Immediately after extraction, the teeth were rigorously cleaned to remove remnants of soft tissue, blood and bacterial biofilm, then they were examined for possible cracks or other defects (carious lesions or hypoplasia). After the cleaning procedure, the teeth were disinfected in a 0.5% chloramine-T solution and then stored in distilled water at 4 °C.

Standardized Class V cavities were prepared by a single operator on the facial and lingual surfaces of all teeth with 4 mm length, 3 mm height and 2 mm depth, with 90-degree cavosurface angles and uniform depth of the axial line angles, and the gingival margin placed 1 mm below the enamel–cement junction. Pear-shaped fine-grained diamond burs (830L 012F FG) were used for the cavity preparation, under abundant water cooling. The diamond burs were activated by a high-speed handpiece (Woodpecker Medical Instruments Co. Ltd., Guilin, Guangxi, China). The cavities were cleaned and dried without desiccation with the air-water spray of the dental unit.

After cavity preparation, the samples were randomly divided in two groups (I and II) corresponding to the etching protocol that had been used. In each tooth, one cavity was randomly assigned for the control subgroup (restoration applied with adhesive only), and the opposite cavity was assigned for the corresponding study subgroup (with preliminary application of chlorhexidine solution—Gluco CHeX 2% (Cerkamed, Stalowa Wola, Poland). The tooth surfaces where chlorhexidine solution was applied were marked with a groove to distinguish between the control and study subgroups.

In both groups, the cavities were restored using a universal adhesive system (Single Bond Universal Adhesive, 3M-ESPE, St. Paul, MN, USA) and a bulk-fill composite resin (Filtek One Bulk Fill Restorative, 3M-ESPE, St. Paul, MN, USA). The adhesive and the bulk-fill composite resin composition are described in Table 1.

According to the etching strategy and chlorhexidine application, four subgroups of specimens resulted, as described in Table 2.

In group I, the adhesive system was applied using the etch-and-rinse technique. In subgroup IA, cavities were etched with 37% phosphoric acid (Etching Gel Jumbo, Kerr Restoratives, Orange, CA, USA) on both dentin and enamel for 15 s, then abundantly washed with water [26]. The pooling water was removed with a cotton roll. In subgroup B, after acid etching and washing, a 2% chlorhexidine solution was applied into the cavity using a soaked cotton roll and left for 60 s [27]. Afterwards, the excessive solution was absorbed with a dry applicator. In both subgroups, the adhesive resin was applied to the entire prepared surface for 20 s [28]. Afterwards, the cavity was gently air-dried for 5 s, then the adhesive was light-cured using a light-curing lamp (X-Cure Led, WoodPecker, Guilin, Guangxi, China) for 20 s, according to the manufacturer’s instructions.

In group II, the same adhesive system was applied using the self-etching technique. In subgroup IIA, the adhesive resin was applied according to the manufacturer’s instructions. In subgroup IIB, a 2% chlorhexidine solution was applied for 60 s, using the same application protocol as in subgroup IB, prior to the application of the adhesive resin.

All cavities were immediately filled using a bulk-fill composite resin applied in a single-layer technique and light-cured for 40 s, according to manufacturer instructions. The external surfaces of each sample were covered with two layers of acid-resistant nail varnish, except for the restoration, and a distance of about 1 mm around the tooth-restoration interface.

### 2.2. Pulpal Pressure Simulation

After the cavity filling, the roots of each tooth were removed 3 mm below the enamel–cementum junction with a diamond disc (Disc DS022, Clinique, manufacturer information is not provided), under continuous water cooling.

Then, the pulp tissue was removed using small excavators, and a 17% EDTA solution (Endo-Solution, Cerkamed, Stalowa Wola, Poland) was applied to the pulp chamber for 1 min. Then the pulp chamber was thoroughly rinsed with distilled water.

Each tooth fragment was fixed using a cyanoacrylate adhesive (Loctite, Westlake, OH, USA) to the inner face of the metal cap of a glass container. Through the cap, a 3 mm high syringe needle was inserted into the pulp chamber. The needle was connected to a perfusor coupled to a 100 mL saline solution vial. The vial was fixed at a height of 20 cm to produce a hydrostatic pressure of 20 cm H_2_O in the pulp chamber [29,30].

### 2.3. Simulation of Cariogenic Attack

To simulate a cariogenic attack, the storage containers were filled with 20 mL of demineralizing solution for 3 days at a constant temperature of 25 °C. The composition of the demineralizing solution was: 0.2 M lactic acid; 3.0 mM CaCl_2_; 1.8 mM KH_2_PO_4_, at a pH of 4.5 [31]. The pH was assessed every 12 h using a portable pH meter (Thermo Scientific Eutech pH 5+, Vernon Hills, IL, USA).

### 2.4. Samples Preparation for the Microleakage Study

The demineralized solution was replaced with distilled water, the specimens were rinsed with distilled water and then they were re-immersed in distilled water for 24 h and then in 1% methylene blue (Vitalia Pharma, Ploiesti, Romania) for 24 h. Finally, the specimens were cleaned under running water for 5 min and then removed from the storage containers. The teeth were sectioned through the middle of the restoration in a facial–lingual direction, using thin diamond discs (Disc DS022, Clinique) with low speed and continuous water cooling in order to reduce the risk of damage. The slices were finished and polished using the Sof-Lex Finishing and Polishing Kit (Batch No. NC11462, 3M ESPE, St. Paul, MN, USA). The cross-sections were ultrasonically cleaned in deionized water for 10 min in order to remove the smear layer produced by the cutting procedures.

The images of the microleakages at the occlusal and cervical margins were registered and scored using an optical Carl-Zeiss AXIO Imager A1m microscope, (Carl-Zeiss, Jena, Germany) coupled with a high-resolution digital camera, capable of obtaining images between 50 and 1000×, using Dark Field and Bright Field filters.

Dye penetration was evaluated according to a 4-point scale: 0 = no dye penetration; 1 = dye penetration from the cavosurface margin to less than half the length of the prepared wall; 2 = dye penetration from the cavosurface margin to more than half the length of the prepared wall, but not involving the axial wall; 3 = dye penetration from the cavosurface margin along the whole length of the prepared wall and also involving the axial wall. The evaluations were carried out in a blind study to overcome the subjectivity of reading.

The tested hypothesis sustained the absence of statistically significant differences between microleakage scores of the study subgroups and corresponding control subgroups for both etching strategies.

### 2.5. Samples Preparation for the SEM Study

The morphology of the interface between the dentin and the composite resin was analyzed using a SEM Vega Tescan LMH II scanning electron microscope (Tescan, Kohoutovice, Czech Republic) with 30 kV and 15.5 WD operating conditions. The cervical and occlusal margins and internal walls were observed in terms of integrity and microgaps formation at different magnifications. The samples were coated with a gold 10 nm layer using a LUXOR^TM^ benchtop sputter coater (ULVAC Technologies, Inc., Kanagawa, Japan).

### 2.6. Statistical Analysis

For the statistical analysis, IBM SPSS 26.0 (SPSS Inc., Chicago, IL, USA) software was used. The Shapiro–Wilk test was used to test the normal distribution of the data, and the statistical analysis was performed using the Kruskal–Wallis test at a significance level of 95%.

## 3. Results

### 3.1. Microleakage Evaluation

**Figure 1:**
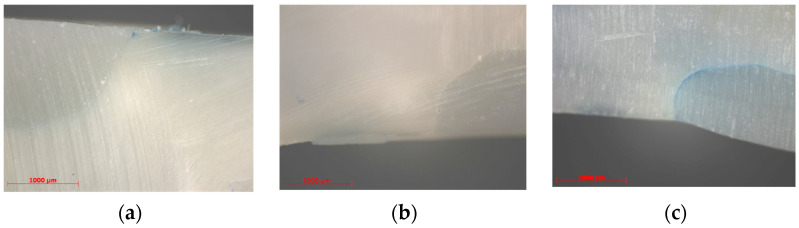
Optic microscope images of microleakage at cervical interface (50 X magnification). (**a**) Cervical microleakage score 1 subgroup B; (**b**) Cervical microleakage score 0 subgroup D; (**c**) Cervical microleakage score 3 subgroup D.

In subgroup A, the majority of the samples presented a score of 2 at the cervical and 1 at the occlusal interface. In subgroup B, most samples presented a score of 1 at both the cervical and occlusal interfaces. In subgroup C, the samples recorded with preponderance a score of 1 at the cervical and 0 at the occlusal interface, and in subgroup D, more than half of the samples presented a score of 1 at both the cervical and occlusal interfaces (Figure 1).

Examples of images showing the microleakage, evaluated with scores of 0, 1 and 3 at the cervical interface, for some samples in subgroups B and D are presented in Figure 1a–c.

**Figure 2:**
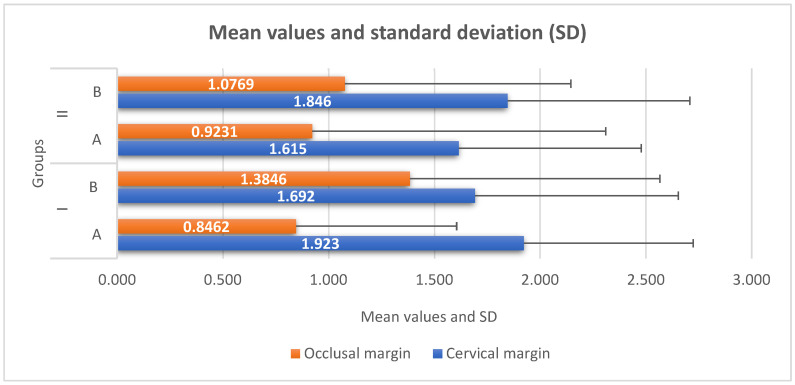
Mean values and standard deviation at occlusal/cervical margin for each group. IA—etch-and-rinse technique (adhesive only); IB—etch-and-rinse technique (2% clorhexidine solution pre-treatment); IIA—self-etch technique (adhesive only); IIB—self-etch technique (2% chlorhexidine solution pre-treatment).

The mean values and standard deviation (SD) of the microleakage test at the occlusal and cervical margins are presented in Figure 2. For the cervical margin, the highest mean value was recorded by subgroup IA (1.923), followed by subgroups IIB (1.846), IB (1.692) and IIA (1.615). For the occlusal margin, subgroup IB reached the peak, with a mean value of 1.385, followed by subgroups IIB (1.077), IIA (0.923) and IA (0.846).

Analyzing the values recorded by each group for the cervical and occlusal margins, it can be observed that there are no statistically significant differences between the subgroups (Table 3).

The statistical results show that for subgroup IA, between the values obtained at the cervical vs. occlusal margins, statistically significant differences were recorded, *p* = 0.001. The mean rank for the cervical margin is 1.92, while the mean rank for the occlusal margin is 0.84, which shows that the values from the cervical margin tend to be higher than those of the occlusal margin (Table 4).

### 3.2. Scanning Electron Microscopy Evaluation

**Figure 3:**
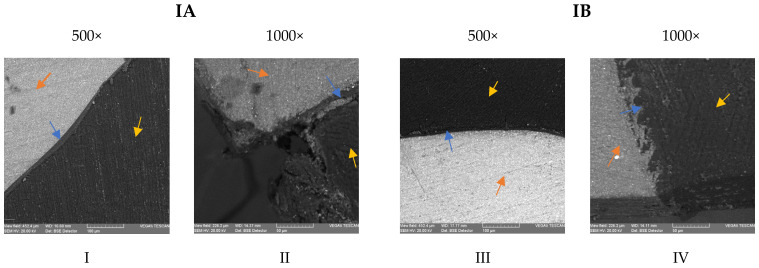

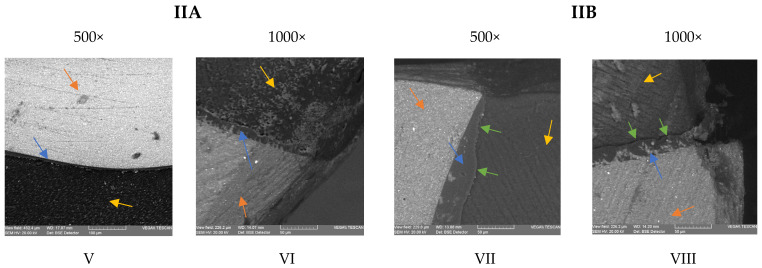
SEM micrographs of the resin–dentin interface after etch-and-rinse technique (**IA**); after etch-and-rinse and 2% chlorhexidine solution application (**IB**); after self-etch technique (**IIA**); after self-etch and 2% chlorhexidine solution application (**IIB**); at 500× and 1000× magnification. * → composite resin; → dentin; → adhesive layer; → marginal defects.

In subgroup IA, a tight contact between the composite resin and the dentin, mediated by a consistent layer of adhesive resin, was observed in most images (Figure 3I). At the cervical margin, adjacent cement/dentin loss and marginal gaps extending up to 50 μm were observed in several sections. Deterioration of the marginal adaptation was observed, with the remaining adhesive resin layer attached to the composite and not sealing the dentin. (Figure 3II). In subgroup IB, thinner layers of adhesive resin were observed at the interface with the dentinal walls (Figure 3III). The sealing of dentin was adequate in most of the images, even at the cervical margins, with preservation of the hybrid layer at the interface (Figure 3IV).

Regarding the dentin interface for subgroup IIA, an intimate contact between the dentin and the composite material was observed on the cavity walls, mediated by a thin and consistent layer of adhesive resin (Figure 3V,VI). For subgroup IIB, the adhesive resin layer was uneven in thickness, but tightly sealed the walls and the margins of the cavities in most images (Figure 3VII). Some minor marginal defects were observed; still, the adhesive layer remained attached on the cementum/dentin margin, despite the deterioration of the root cementum (Figure 3VII,VIII).

## 4. Discussion

After polymerization, the residual water from dentin is the source for the hydrolytic degradation processes of both the adhesive resin and the demineralized collagen [32]. Therefore, it seems likely that simulating pulpal pressure will have a negative impact on the interface when samples are artificially aged [33].

However, it can be considered that pulp pressure simulation during sample aging could provide a more appropriate strategy to assess the real performance of adhesive systems and composite resin restorations in vitro. Feitosa et al. support this hypothesis, showing that it was possible to achieve faster aging of the resin–dentin interface by using simulated pulp pressure, although this technique was only used after adhesive application [33].

This study aimed to use a similar protocol for simulating pulpal pressure, combined with storage of the samples in a demineralizing solution, to simulate an acute cariogenic attack [28]. This demineralizing solution was firstly used by Matsuda et al. in an automatic pH-cycling system and later by Yagi et al. [31,34]. The latter study used storage for 3 days in this demineralization solution to measure Ca and F distributions and concentrations in a root caries model. Other similar studies used distilled water or artificial saliva as a storage medium [35,36]. The use of a demineralizing solution as a storage medium simulates more accurately the behavior of the adhesive interface in the oral environment [12].

Chlorhexidine gluconate is one of the most common disinfectants used in caries treatment. Above the antibacterial properties, chlorhexidine exerts several effects on dentin, which might affect its structure and properties, including the bonding and interface with composite resins. The symmetrical chlorhexidine molecule has two positive charges that mediate electrostatic attraction to the phosphate anions in the hydroxyapatite structure to form crystals [2,37,38,39]. The application of a 2% chlorhexidine solution on dentin resulted in precipitates formation and changes of the morphology and chemical properties of the dentin that could affect the bonding strength [2,7].

Furthermore, it has been suggested that dentin becomes resistant to acid etching due to chlorhexidine residues [40], and this acid-resistant layer could prevent the infiltration of hydrophilic resin into dentin [39]. Several studies demonstrated that the hybrid layer formed within the dentin treated with chlorhexidine is thinner, less uniform and with fewer resin plugs [9,39,40], which is consistent with the SEM images that we have registered during our study. Furthermore, there is evidence that CHX inhibits the activity of MMPs and cysteine cathepsins [41], which might reduce the susceptibility to degradation of the hybrid layer.

The images recorded by the scanning electron microscope suggest that 2% chlorhexidine application might have positively influenced the quality of the interface at the dentin–cementum margins when the universal adhesive was used in the etch-and-rinse strategy. The results are in accordance with previous studies by Breschi et al., in which etched dentin subsequently treated with 2% chlorhexidine showed an increased bond strength and a better quality of the hybrid layer compared to the control groups [37]. Moreover, the acid-base resistant zone created by self-etch adhesives could not be observed when protocols involving etching with phosphoric acid were used, although the hybrid layers were thicker [42]. The storage in the acidic solution resulted in more pronounced alterations of the cementum–dentin margins in several specimens of subgroup IA, comparing to subgroup IB, where dentin resistance to acid challenge had been increased due to chlorhexidine residues. It seems that when the etch-and-rinse strategy is used, the chlorhexidine-impregnated layer could compensate for the absence of the acid-base resistant zone, at least for short-term acidic challenge. Contrary to our findings, Kimyai et al. showed that the use of chlorhexidine application increased the size of the gingival margin gaps, irrespective of the bonding strategy applied, when distilled water storage and thermocycling was used for simulating the oral environment [40]. These contradictory results could be explained by the different properties of the tested adhesives, but mainly by the impact of the aging strategy on the hydrolytic degradation of the interface at the margins [34]. In our study, the marginal adaptation was influenced not only by the storage in demineralizing solution, but also by the high configuration factor of the cavity and the pulpal pressure simulation, which can result in higher hydrolytic degradation of the resin–dentin interface [29].

On the other hand, the same acid-resistant layer created by chlorhexidine could be responsible for detrimental effects on the sealing of dentin/enamel margins observed for self-etch adhesive systems. The dentin becomes resistant to etching when a mild self-etch adhesive is used due to chlorhexidine residues [40], and this acid-resistant layer could prevent the infiltration of hydrophilic resin into dentin [42]. This might explain the uneven thickness of the adhesive resin layer and minor marginal defects that have been registered in several images of subgroup IIB.

Moreover, the tested universal adhesive (Single Bond Universal Adhesive, 3M-ESPE) contains MDP and HEMA, which may result in incompatibility with chlorhexidine and sensitivity to pulpal pressure simulation [5]. 10-MDP (10-Methacryloyloxidecyl dihydrogen phosphate) can form a chemical bond with the hydroxyapatite calcium around the collagen fibers [40,43,44]. Chlorhexidine’s reaction with dentinal calcium results in lower levels of calcium available for binding with 10-MDP; therefore, the adhesive cannot form an adequate bond with the tooth structures [40,45].

A recent study tested the same universal adhesive system, with and without preliminary chlorhexidine application, in self-etch and etch-and-rinse strategies after storage in distilled water for 30 days. The etch-and-rinse technique resulted in uniform hybrid layers, while the pretreatment with chlorhexidine 0.2% created thinner and less uniform hybrid layers. When the adhesive was applied in the self-etch strategy, the thicknesses of the hybrid layers were smaller, but uniform, and the formation of resin plugs was not observed. When 0.2% chlorhexidine was preliminary applied, no regions with evident and uniform hybrid layers were observed [40]. These results are similar to our findings, except for subgroup IIB (self-etch technique and chlorhexidine), where the images showed uneven, but consistent adhesive resin layers. The deterioration of the cervical margins that was recorded in our images are probably related to the acidic challenge determined by the storage of the specimens in demineralization solutions.

Since the SEM images seemed to suggest some morphological differences of the adhesive interface mainly in the cervical margins, it would have been expected that the microleakage values would be significantly different between the study groups and the control groups. The mean value of the microleakage in the cervical margin was higher for subgroup B (1.69 ± 0.77), comparing to subgroup IA (1.92 ± 0.72), and lower for subgroup IIA (1.61 ± 0.92), comparing to subgroup IIB (1.84 ± 0.94). These results seem to support the hypothesis that chlorhexidine could impair an adequate bonding of the adhesive when used in the self-etch strategy and improve the bonding quality of the adhesive applied in the etch-and-rinse strategy. However, as regarded the microleakage scores at the cervical margin, there were no statistically significant differences between the groups, not even in the case of subgroup IA, where the SEM images recorded the deterioration of the interface at the cervical margins in several samples. It seems that these changes involved mostly the superficial dentin, and they were apparently insufficient to compromise the sealing in deeper areas. The morphological differences observed at the interface did not significantly influence the sealing capacity of the tested adhesive, regardless of the etching strategy. Further research is needed to evaluate this effect in the case of aging samples for longer periods when combining pulp pressure simulation and pH-cycling.

The microleakage scores were higher at the cervical margins compared to the enamel margins for all tested groups; however, the difference was statistically significant only in control subgroup IA. These findings are consistent with the results of previous studies supporting the superiority of bonding to enamel when phosphoric acid is used for etching and the difficulties of bonding to enamel for mild self-etch adhesives [39,40].

Other studies evaluated the influence of pretreatment with chlorhexidine on marginal microleakage [46,47,48,49]. The preliminary application of a 2% chlorhexidine solution determined significantly higher microleakage at the gingival margin with a seventh-generation adhesive, even if the detrimental effects were lower than when using other disinfectants (2.5% sodium hypochlorite and 2% iodine) [46]. In the case of the same adhesive and composite material that we tested in our study, Bin Shuwais and colleagues concluded that disinfection with chlorhexidine decreased microleakage when the etch-and-rinse strategy was applied and did not significantly affect the marginal sealing in the self-etch protocol. In all groups, the cervical margins showed significantly higher microleakage than did the occlusal margins [50].

One limitation of the study is represented by the evaluation of the bonding capacity of universal bonding systems only by analyzing the morphological aspect of the adhesive interface and microleakage assessment. Further studies are needed to evaluate the long-term bonding strength under pH-cycling conditions, as well as testing the bonding capacity of the chlorhexidine combination with other adhesive systems. Moreover, pulp pressure stimulation only after bonding application can be considered another limitation of the study. In the conditions of the present study, it can be appreciated that under conditions of pulp pressure and caries attack simulation, the pre-treatment with chlorhexidine did not affect the quality of the dentin sealing, regardless of the etching protocol.

## 5. Conclusions

Application of chlorhexidine on tooth substrate before using a universal bonding system in total-etch or self-etch mode has no influence on the adhesive interface in conditions of cariogenic attack. The thickness of the adhesive resin layer seems to be less uniform when using chlorhexidine, but the morphological differences at the adhesive interface have no influence on the sealing capacity of the universal bonding system, regardless of the etching strategy.

## Figures and Tables

**Table 1:** Composition of the materials.

Name	Manufacturer	Type	Batch no.	Composition
Single Bond Universal	3M ESPE, St. Paul, MN, USA	Universal Adhesive	7601156	10-MDP phosphate monomer, Vitrebond copolymer, HEMA, dimethacrylate resins, filler, silan, initiatiors, ethanol, water
Filtek One Bulk Fill Restorative	3M ESPE, St. Paul, MN, USA	Bulk Fill composite resin	NC90177	Bis-GMA, UDMA, Bis-EMA, TEGDMA, EDMAB, silica/zirconia, YbF_3_

10-MDP—10-Methacryloyloxydecyl dihydrogen phosphate; HEMA—Hydroxyethyl methacrylate; Bis-GMA—Bisphenol A diglycidyl ether methacrylate; UDMA—Urethane dimethacrylate; Bis-EMA–ethoxylated bisphenol-A dimethacrylate; TEGDMA—Triethylenglycol dimethacrylate; EDMAB—ethyl 4-dimethyl aminobenzoate.

**Table 2:** Study groups.

GROUP	SUBGROUP						
I	A	Control group (n = 20)	Etch-and-rinse technique	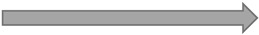			Adhesive
	B	Study group (n = 20)	Etch-and-rinse technique	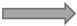	Pretreatment with 2% Chlorhexidine solution	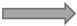	Adhesive
II	A	Control group (n = 20)	Self-etch technique	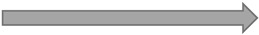			Adhesive
	B	Study group (n = 20)	Self-etch technique	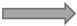	Pretreatment with 2% Chlorhexidine solution	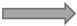	Adhesive

**Table 3:** Statistical differences between groups for occlusal/cervical margin.

Oclusal					Cervical				
subgroups					subgroups				
	IA	IB	IIA	IIB		IA	IB	IIA	IIB
IA	-	* 0.416	* 0.816	* 0.629	A	-	* 0.731	* 0.748	* 0.867
IB	* 0.416	-	* 0.451	* 0.399	B	* 0.731	-	* 0.859	* 0.813
IIA	* 0.816	* 0.451	-	* 0.653	C	* 0.748	* 0.859	-	* 0.639
IIB	* 0.629	* 0.399	* 0.653	-	D	* 0.867	* 0.813	* 0.639	-

* Not statistically significant *p* < 0.05. IA—etch-and-rinse technique (adhesive only); IB—etch-and-rinse technique (2% clorhexidine solution pre-treatment); IIA—self-etch technique (adhesive only); IIB—self-etch technique (2% chlorhexidine solution pre-treatment).

**Table 4:** Statistical differences between the values obtained at cervical and occlusal margins in each subgroup.

Subgroups	IA	IB	IIA	IIB
	Cervical			
Occlusal	** 0.001	* 0.473	* 0.143	* 0.075

* Not statistically significant. ** Statistically significant *p* < 0.05. IA—etch-and-rinse technique (adhesive only); IB—etch-and-rinse technique (2% clorhexidine solution pre-treatment); IIA—self-etch technique (adhesive only); IIB—self-etch technique (2% chlorhexidine solution pre-treatment).

## Data Availability

Not applicable.
